# Evaluation of NAQFC model performance in forecasting surface ozone during the 2011 DISCOVER-AQ campaign

**DOI:** 10.1007/s10874-013-9251-z

**Published:** 2013-03-09

**Authors:** Gregory G. Garner, Anne M. Thompson, Pius Lee, Douglas K. Martins

**Affiliations:** 1 Department of Meteorology, The Pennsylvania State University, 503 Walker Building, University Park, PA 16802 USA; 2 National Oceanic and Atmospheric Administration USA, Air Resources Laboratory, College Park, MD USA

**Keywords:** Air quality, Model evaluation, CMAQ, NAQFC, DISCOVER-AQ, Ozone

## Abstract

The National Air Quality Forecast Capability (NAQFC) and an experimental version of the NAQFC (NAQFC-*β*) provided flight decision support during the July 2011 NASA DISCOVER-AQ field campaign around Baltimore, Maryland. Ozone forecasts from the NAQFC and NAQFC-*β* were compared to surface observations at six air quality monitoring stations in the DISCOVER-AQ domain. A bootstrap algorithm was used to test for significant bias and error in the forecasts from each model. Both models produce significant positively biased forecasts in the morning while generally becoming insignificantly biased in the afternoon during peak ozone hours. The NAQFC-*β* produces higher forecast bias, higher forecast error, and lower correlations than the NAQFC. Forecasts from the two models were also compared to each other to determine the spatial and temporal extent of significant differences in forecasted ozone using a bootstrap algorithm. The NAQFC-*β* tends to produce an average background ozone mixing ratio of at least 3.51 ppbv greater than the NAQFC throughout the domain at 95 % significance. The difference between the two models is significant during the overnight and early morning hours likely due to the way the Carbon Bond 5 mechanism in the NAQFC-*β* handles reactive nitrogen recycling and organic peroxide species. The value of information each model provides was tested using a static cost-loss ratio model. By standard measures of forecast skill, the NAQFC generally outperforms the NAQFC-*β*; however, the NAQFC-*β* provides greater value of information. This is because standard measures of forecast skill often hide the sensitivity of end users’ needs to forecast error.

## Introduction

The NASA Earth Venture Program on **D**eriving **I**nformation on **S**urface conditions from **CO**lumn and **VER**tically resolved observations relevant to **A**ir **Q**uality (DISCOVER-AQ) used a combination of aircraft and ground stations to assess the air quality around Baltimore, Maryland, in July 2011. A team was tasked with providing meteorological and air quality forecasts in support of safe and project-effective flight operations for the two research aircraft. The team provided briefings, which included detailed 24-h forecasts, extended 5-day outlooks, and flight recommendations. With the cost of flight-hours reaching $45,000 per day, it is important to optimize flight decisions using state-of-the-art prognostic tools.

Among the numerous sources of information and tools used to prepare these briefings were two numerical air quality models. One of the numerical models was the National Air Quality Forecast Capability (NAQFC), the current national operational air quality model that provides forecasts of surface ozone and smoke. The other numerical model was an experimental version of the NAQFC (NAQFC-*β*), which provided forecasts of particulate matter in addition to the current capabilities of the operational model. The NAQFC-*β* was provided by the NOAA Air Resources Laboratory to help address a secondary objective of the DISCOVER-AQ campaign, which was to evaluate state-of-the-art air quality models. The combination of these air quality models and the unique flight-decision support needed during DISCOVER-AQ yielded an ideal test-bed for addressing the value of information in real-time decision scenarios.

The questions this work seeks to answer pertain to the secondary objective of DISCOVER-AQ, model evaluation. First, how do each of the numerical air quality models perform during the DISCOVER-AQ campaign? Second, how do the underlying differences between the two models impact the forecasted air quality? Finally, what do these differences mean to the end user in terms of the value of information?

## Background

The NAQFC is an offline system consisting of the Weather Research and Forecasting Non-hydrostatic Meso-scale meteorological Model (WRF-NMM) (Janjic 2003) coupled with the Community Multi-scale Air Quality Model (CMAQ) (Byun and Schere 2006) that has been providing forecasts of near-surface ozone since 2005. Forecasts produced by the NAQFC have been rigorously verified with observations (Ryan et al. 2004; Eder et al. 2006, 2009, 2010). The NAQFC is generally biased high when forecasting summertime surface ozone in urban areas of the eastern United States by as much as 5.5 ppbv. Despite the bias, the NAQFC was found to produce valuable forecasts for relatively low to moderate cost decision scenarios, especially in Washington, DC, and Baltimore, MD (Garner and Thompson 2012).

The NAQFC-*β* differs from the NAQFC by upgrading CMAQ from a gas-phase-mechanism (version 4.5) to a full gas and aerosol mechanism (version 4.6) while remaining similar in all other aspects (Lee and Ngan 2011). CMAQ model version 4.6 is based on the Carbon Bond 2005 (CB05) gas-phase chemical mechanism (Yarwood et al. 2005) with modal size-distributed aerosol components (Foley et al. 2010). Both the NAQFC and NAQFC-*β* are run with the same meteorological fields, emissions inventories, and horizontal grid spacing of 12 km.

Area and mobile emissions are based on the EPA National Emissions Inventory (NEI) for 2005. For Electric Generating Unit (EGU) point sources, Continuous Emission Monitoring 2009 replaces the 2005 NEI where applicable. Updated EGU emissions are further projected into 2011 using emission projection factors from the Department of Energy 2011 Annual Energy Outlook report (http://www.eia.gov/forecasts/archive/aeo11). All emissions that are independent from meteorological conditions are processed prior to the model execution using a modified version of the Sparse Matrix Operator Kernel Emission (SMOKE) model (Houyoux et al. 2000). Emissions dependent on meteorological conditions are simulated during model execution through a CMAQ-pre-processor. Monthly mean lateral boundary conditions adopted from a species mapping methodology are incorporated into the models (Tang et al. 2008). A selected set of chemical fields derived from the GEOS-CHEM global model simulation with assimilated meteorology for July 2006 is employed (Bey et al. 2001).

The CB05 mechanism used in the NAQFC-*β* tends to produce 2.0–5.0 ppbv more ozone than the CB-IV mechanism used in the NAQFC (Sarwar et al. 2008) in the Mid-Atlantic region. Sensitivity tests on the CB05 mechanism found that additional reactions associated with organic peroxide species and reactive nitrogen recycling are primarily responsible for the additional ozone (Saylor and Stein 2012). Although these results are useful, how these differences impact the utility of these models to the end user has been unknown until examined in detail here.

## Data

Hourly average surface ozone forecasts for each day in July 2011 from both the NAQFC and the NAQFC-*β* were used in this statistical analysis. Forecasts were taken from the 1200 UTC model runs from the previous day since it is these forecast fields that air quality forecasters and decision makers use. The Maryland Department of the Environment (MDE) provided 1-h average near-surface ozone mixing ratios as measured by UV photometers at the six monitors of interest during the campaign (Table 1). These observations are used to verify the 1-h average surface ozone mixing ratio forecasts produced by both the NAQFC and the NAQFC-*β* from the previous day.

**Table 1:** Locations and descriptions of the six surface monitors involved in the DISCOVER-AQ campaign

Site Name	FIPS Code	Type	Lat [deg N]	Lon [deg E]	Elev. [m]
Aldino	240259001	Commercial Suburban	39.563	−76.204	127.711
Beltsville	240330030	Residential Suburban	39.028	−76.817	52.730
Edgewood	240251001	Military Rural	39.410	−76.297	8.534
Essex	240053001	Residential Suburban	39.311	−76.474	12.802
Fairhill	240150003	Residential Rural	39.701	−75.860	117.652
Padonia	240051007	Residential Suburban	39.461	−76.631	119.481

**Fig. 1:**
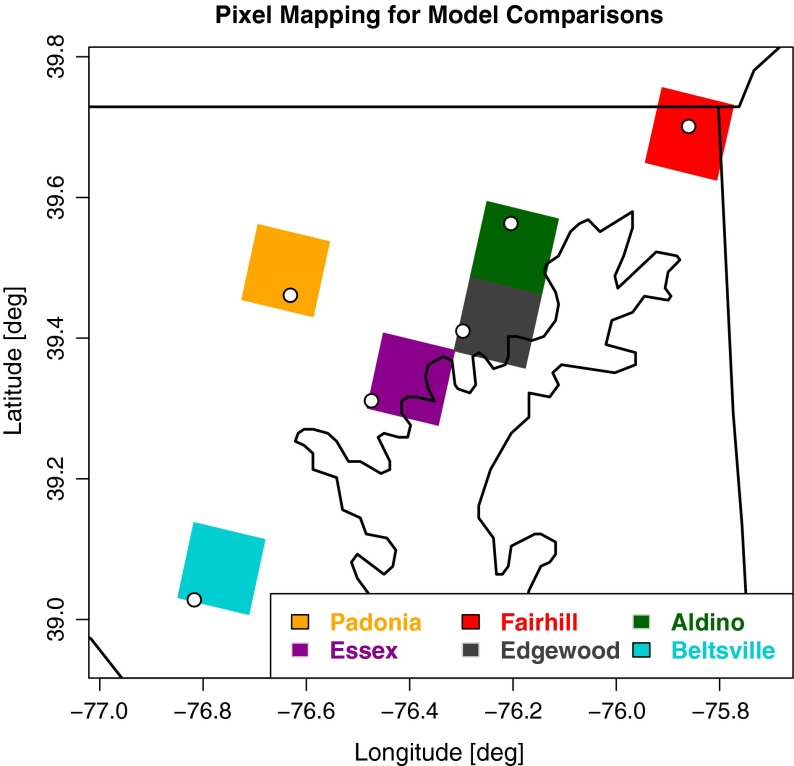
Map of the MDE monitor locations and model pixels used in the analysis. Pixels are color-coded according to the corresponding monitor. The monitor locations are indicated by the white dots within the model pixels

Figure 1 identifies the MDE monitor locations along with the nearest model pixels used in this analysis. The monitors generally lie near the periphery of the model grid points, with the exception of Fairhill, and the model grid points associated with Edgewood and Essex contain a significant amount of water from the Chesapeake Bay. Though the peripheral location of the monitors may induce small representation errors, the mixed land-water pixels at Edgewood and Essex may induce notable features in the performance metrics.

Decisions made in air quality management are generally associated with pollution exceedances with reference to the National Ambient Air Quality Standards (NAAQS). The primary NAAQS regarding surface ozone of 75 ppbv is based on a daily maximum of forward running 8-h average mixing ratios. Thus all ozone mixing ratios, both MDE observed and model predicted, have been averaged as such. For any given hour, the current 1-h average ozone mixing ratio was arithmetically averaged with the subsequent 7 hours of the 1-h average ozone mixing ratios. Averages calculated with no more than 2 hours missing were included in the analysis.

## Methods

### Bootstrapping

A bootstrap sampling algorithm (Efron 1979; Efron and Gong 1983), or “bootstrapping”, is used throughout this work to test for significant differences between the NAQFC and the NAQFC-*β*. Bootstrapping involves repeatedly sampling a dataset with replacement in order to account for sampling uncertainties. The original dataset is considered a pool of samples from which bootstrap sub-samples are drawn. A statistical metric (i.e. bias, error, correlation, etc.), a model, or any other sort of analysis is then performed on the bootstrap sub-sample. Once the analysis is complete on this bootstrap sub-sample, another bootstrap sub-sample is drawn. This process continues for a user-defined (and often large) number of iterations.

Bootstrapping produces a distribution of results from the analyses performed on each bootstrap sub-sample. Various descriptive statistics (such as mean, median, quantiles, etc.) are then used to describe the distribution about the metric which in turn are analogous to the uncertainties about the metric of the original dataset. Often times, the 2.5 % and 97.5 % quantiles of the distribution are used to empirically represent the 95 % confidence interval (CI) about the given metric. A quantity would then be considered significantly different from a given value if that value is not contained within the CI. Differences between two sources of data can be tested for significance by bootstrapping the same metric from each of the two sources and comparing the resulting CI from each distribution. Distributions of the same metric for different sources are considered significantly different if the CIs do not overlap.

To test for significant spatial and temporal differences between the NAQFC and NAQFC-*β*, bootstrapping was applied to each forecast hour in each pixel of the co-located model domain. For any given pixel and hour of the day, the 31 daily forecasts comprised the sample pool from which 10,000 bootstrap subsamples were produced. The *CI* about the mean residual, referenced to the NAQFC (i.e. NAQFC-*β* − NAQFC), were calculated from each bootstrap subsample. Insignificant differences will contain zero within the bounds of the *CI*. The minimum difference to insignificance (MDI) is defined as the *CI* bound closest to zero in those cases where the differences are significant or

1 $$ MDI = \begin{cases} CI_{lo} & \text{if $CI_{lo}>0$,}\\ CI_{up} & \text{if $CI_{up}<0$,}\\ 0 & \text{if $0 \in CI$} \end{cases} \label{eq:MDI} $$

where *CI*_*up*_ and *CI*_*lo*_ are the upper and lower bounds of *CI* respectively. The lower CI bound is used when the difference is positive (NAQFC-*β* > NAQFC) and the upper CI bound is used when the difference is negative (NAQFC-*β* < NAQFC).

Model bias and error were tested for significance at each site in Fig. 1 (Table 1) using bootstrapping. For each hour of the day, the 31 available model forecasts and observations for that hour were used as the bootstrap sample pool. The mean bias (MB) and root-mean-squared-error (RMSE) were calculated for each of the 10,000 bootstrap subsamples taken from the sample pool. These metrics are often used to evaluate the performance of a continuous forecast system. Additionally, the difference between the NAQFC and NAQFC-*β* with respect to MB and RMSE were calculated to identify times of the day where the models significantly differ from one another.

### Value of information

A static cost-loss ratio model was used to quantify the value of information that forecast models provide (Thompson 1952; Thompson and Brier 1955; Katz and Murphy 1997; Richardson 2000). This statistical model directly relates various aspects of forecast skill to potential savings in expenditure. For this simple model, consider a decision scenario that requires an action based on the predicted mixing ratio of surface ozone. Let *θ* represent the threshold of surface ozone on which the action depends. Forecasted ozone greater than *θ* would constitute a forecasted ozone event and require a different action than when the forecasted ozone is less than *θ*. Let Θ = ℝ_ > *θ*_ represent all possible values of surface ozone greater than the defined threshold. The forecast hit rate (*Hit*), miss rate (*Miss*), and false alarm rate (*FAR*) are then defined as

2a $$ \label{eq:hit} Hit = \frac{\sum^{N}_{i=1} F_{i} \in \Theta \wedge O_{i} \in \Theta}{N} $$

2b $$ \label{eq:miss} Miss = \frac{\sum^{N}_{i=1} F_{i} \notin \Theta \wedge O_{i} \in \Theta}{N} $$

2c $$ \label{eq:far} FAR = \frac{\sum^{N}_{i=1} F_{i} \in \Theta \wedge O_{i} \notin \Theta}{N} $$

where *F*_*i*_ is the forecasted ozone corresponding to the observed value *O*_*i*_ and *N* represents the total number of forecast-observation pairs. These rates are often used to evaluate the performance of a binary forecast system by providing the frequency at which ozone events were forecasted and observed (*Hit*, Eq. 2a), observed but not forecasted (*Miss*, Eq. 2b), and forecasted but not observed (*FAR*, Eq. 2c).

Now, consider the contingency table (Table 2) that is associated with this simple decision model.

**Table 2:** Contingency table for the simple decision scenario

	*F*_*i*_ > *θ* (Protect)	*F*_*i*_ ≤ *θ* (Do not protect)
*O*_*i*_ ≤ *θ* (Good AQ)	*C*	—
*O*_*i*_ > *θ* (Poor AQ)	*C*	*L*

*F*_*i*_ and *O*_*i*_ represent the forecasted and corresponding observed state of ozone

One would take protective actions if *F*_*i*_ > *θ*, or when the forecasted ozone is greater than a given threshold. This protective action comes at a cost *C* which fully insures against any loss *L* associated with an ozone event whether or not an ozone event occurs. If an ozone event is observed and no protective actions are taken, one would expect to lose *L*. Costs to protect are assumed to be less than the potential losses incurred, otherwise one would never protect and thus there would be no decision to make.

The value of a forecast system can then be defined as

3 $$ \label{eq:value} V = \frac{min(\frac{C}{L},s) - [(Hit+FAR)\frac{C}{L}+Miss]}{min(\frac{C}{L},s) - s\frac{C}{L}} $$

where *s* = *Hit* + *Miss* or the frequency of ozone events that exceed the threshold (Richardson 2000; Garner and Thompson 2012). The first term in the numerator and the denominator represents the expenditures due to the frequency of poor ozone events. The decision-maker would choose to either always protect or incur a loss proportional to the frequency of poor ozone events, whichever expenditure is the least. This provides the baseline from which the value of a forecast system is calculated. The second term in the numerator is the expenditure when using the forecast system. The decision-maker would spend *C* everytime the forecasted ozone is greater than the threshold and incur a loss of *L* everytime poor ozone occurred without being forecasted. The second term in the denominator represents the expenditure of a perfect forecast system. With a perfect forecast system, one would only spend *C* for each poor ozone event and nothing otherwise. Equation 3 has been normalized by *L* to cast value as a function of a cost-loss ratio.

From Eq. 3, value can be interpreted as a savings over no forecast guidance relative to a perfect forecast. The cost-loss ratio can be interpreted as various protective measures from which the decision-maker may choose. With the bounds on *C* mentioned earlier, the cost-loss ratio will range from zero to one with large ratios corresponding to more expensive protective measures. Value is undefined for cost-loss ratios equal to zero or one. A perfect forecast system will have a value of one over all cost-loss ratios between zero and one. Typically, a forecast system will provide value between zero and one over a subset of cost-loss ratios.

To determine the relative value of the models, the static cost-loss ratio algorithm described above is used. The value is first calculated for each of the models separately. The number of hits, misses, and false alarms are counted with reference to the NAAQS for each model and used in Eq. 3. The calculated value of the NAQFC, as a function of the cost-loss ratio, is then subtracted from the calculated value of the NAQFC-*β* to determine the relative difference in value between the two models.

## Results and discussion

### Surface evaluation

**Fig. 2:**
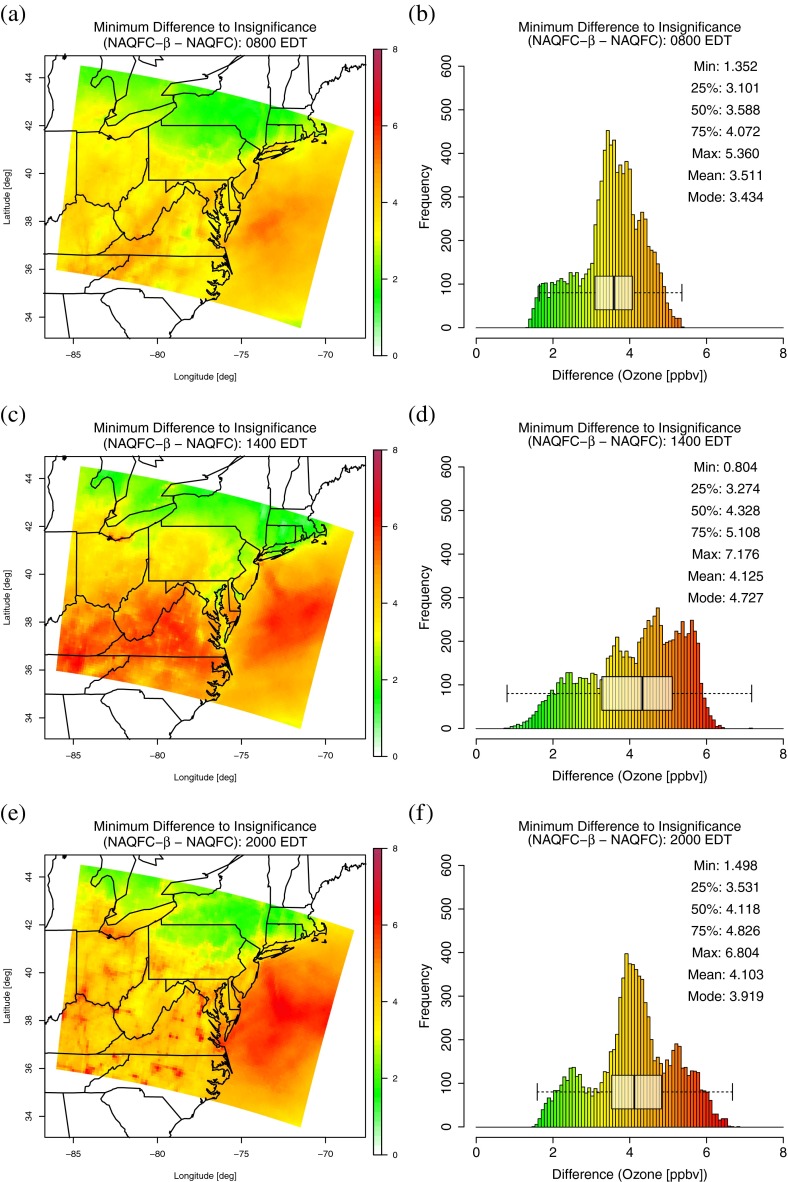
Maps of the MDIin forecasted surface ozone between the NAQFC and the NAQFC-*β* and corresponding histograms with boxplots at **a**, **b**) 0800 EDT, **c**, **d**) 1400 EDT, and **e**, **f**) 2000 EDT. A CI about the mean difference between the two models was calculated for each pixel. The color shading is MDI in ppbv and represents how far the derived CI bounds are from including zero. White pixels indicate insignificant differences. Shades of green, yellow, orange, and red indicate that the NAQFC-*β* is increasingly greater than the NAQFC

Figure 2 contains maps and histograms of the MDI between the NAQFC and NAQFC-*β* at three times of the day.

The MDI between the two models is fairly homogeneous in the morning hours prior to maximum ozone production (Fig. 2a, b). The distribution of the MDI is concentrated about the mean of 3.51 ppbv with an interquartile range of 0.97 ppbv. Large MDI at 0800 EDT (approximately 2–3 ppbv higher than the mean MDI) are observed in large cities, transportation routes, and elevated terrain along the Virginia/West Virginia border. Evidenced by the distribution of MDI and minimal emission impact, it is safe to interpret the background MDI between the two model versions as approximately 3.51 ppbv.

The distribution of MDI in the afternoon is more variable with an interquartile range of 1.83 ppbv (Fig. 2c, d). Many of the features observed in the morning hours are no longer discernible. The elevated MDI appears to be contained within the southern portions of the domain and over the Atlantic Ocean. At 2000 EDT (Fig. 2e, f), the diffuse regional MDI over the land mass is replaced by local maxima collocated with emission sources and transportation routes. The ambient MDI drops back to the background levels similar to those observed in Fig. 2a. Transportation routes, such as interstates, state highways, and waterways, exhibit MDI of approximately 5 ppbv. Large cities are associated with the largest MDI, reaching over 6 ppbv.

The differences observed in the MDI are likely due to the advanced recycling methods of reactive nitrogen in the CB-05 mechanism present in the NAQFC-*β*. The reactive nitrogen recycling effectively converts stable forms of nitrogen into more reactive forms. Incorporating these reactions into the model produce additional pathways through which ozone can be produced, ultimately increasing ozone concentrations.

**Fig. 3:**
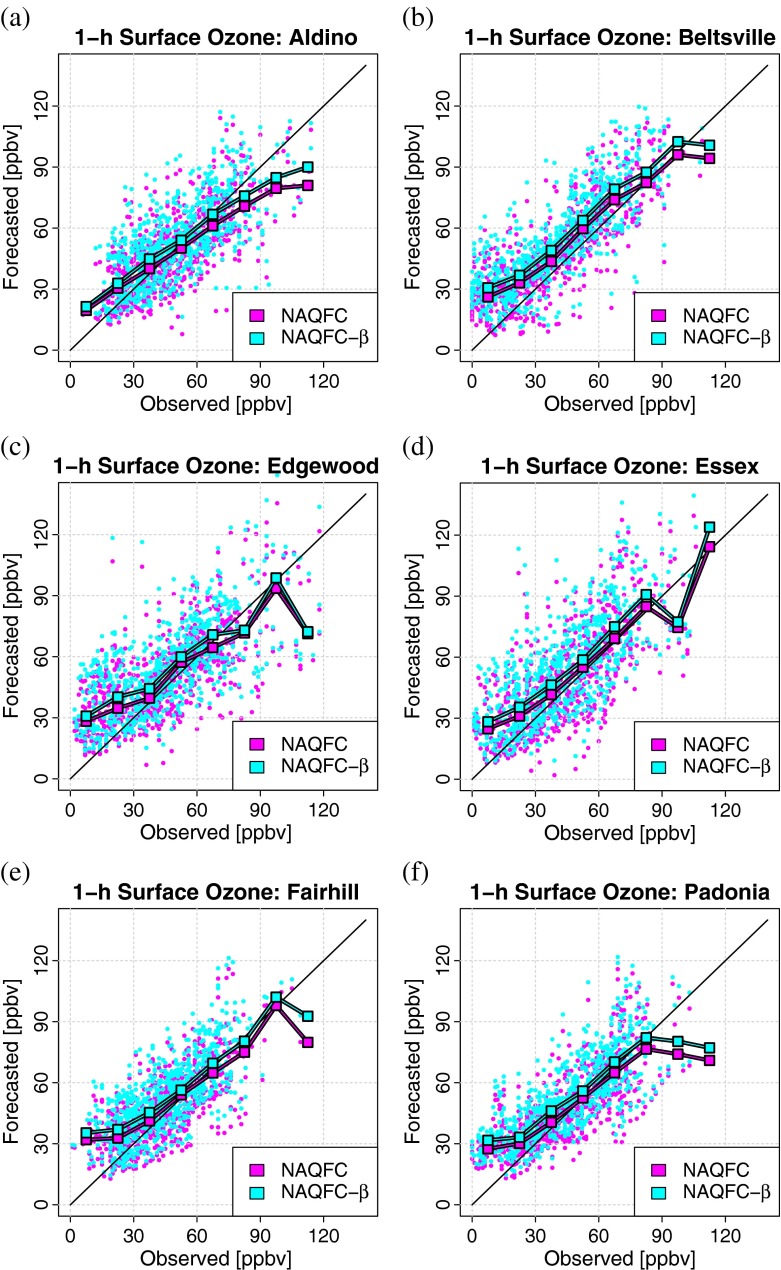
Summary of the general skill of the NAQFC (magenta) and the NAQFC-*β* (cyan) in forecasting 1-h average surface ozone at **a** Aldino, **b** Beltsville, **c** Edgewood, **d** Essex, **e** Fairhill, and **f** Padonia. Each dot represents a single observation-forecast pair. The squares indicate the median forecast for a 15 ppbv bin of observed ozone. The 1:1 line is provided for guidance

Figure 3 and Table 3 describe the general skill of the NAQFC and NAQFC-*β* in forecasting 1-h average surface ozone over the six DISCOVER-AQ sites.

**Table 3:** The statistics calculated from the plots in Fig. 3 including the correlation (Corr.), root-mean-square error (RMSE), mean bias (MB), and the normalized mean bias (NMB) of the forecast models with surface observations

	Corr.		RMSE		MB		NMB	
	NAQFC	NAQFC-*β*	NAQFC	NAQFC-*β*	NAQFC	NAQFC-*β*	NAQFC	NAQFC-*β*
Aldino	0.70^ ∗ ^	0.69^ ∗ ^	15.56	16.39	−1.15	3.40	−2.28 %	6.75 %
Beltsville	0.82^ ∗ ^	0.81^ ∗ ^	16.86	20.12	8.96	13.84	22.34 %	34.49 %
Edgewood	0.69^ ∗ ^	0.67^ ∗ ^	18.75	20.92	4.47	9.03	9.75 %	19.69 %
Essex	0.71^ ∗ ^	0.70^ ∗ ^	18.29	20.66	5.65	10.39	12.88 %	23.67 %
Fairhill	0.73^ ∗ ^	0.72^ ∗ ^	13.59	15.81	3.54	7.81	7.98 %	17.61 %
Padonia	0.77^ ∗ ^	0.77^ ∗ ^	14.39	16.32	3.21	7.78	7.11 %	17.22 %

RMSE and MB are provided in units of ppbv. All statistics are significantly different from zero at the 95 % CI. The relative difference between the NAQFC and the NAQFC-*β* with respect to each of these statistics are also significant with the exception of the correlations(indicated with^ ∗ ^)

The median of the forecasts indicate that overprediction at the lowest observed surface ozone mixing ratios (0–30 ppbv) is common among all six of the sites. The forecasts tend to follow the 1:1 line between 30–75 ppbv of observed ozone mixing ratios. There are too few observation-forecast pairs to definitively say that the models underpredict at high levels of observed ozone (> 75 ppbv), but the plots in Fig. 3 show that this trend is plausible among many of the sites. These descriptive statistics of the comparisons indicate that the NAQFC outperforms the NAQFC-*β*. The differences between the statistics reported for each model were found to be significant with the exceptions being the correlations.

**Fig. 4:**
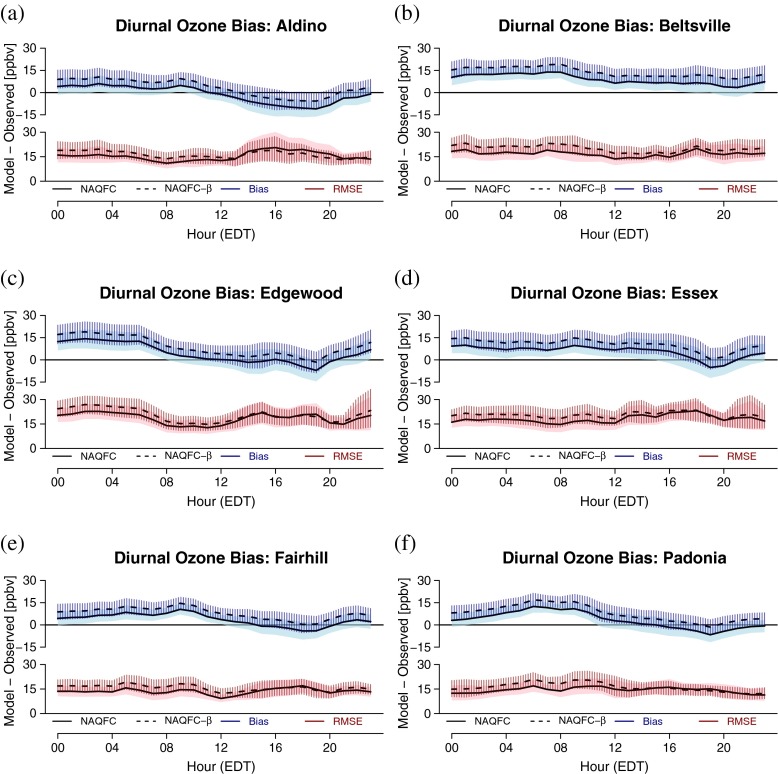
Bias and RMSE as a function of the hour of the day for **a** Aldino, **b** Beltsville, **c** Edgewood, **d** Essex, **e** Fairhill, and **f** Padonia. The model type is denoted by line and fill type (NAQFC - solid; NAQFC-*β* - dashed). The statistic is color-coded (Bias -* blue*; RMSE - *red*). The mean and CI of each statistic is indicated by the line and fill respectively

The bias among all the sites follow a similar diurnal pattern (Fig. 4).

Generally, there is a positive mean bias in the early morning hours that decreases throughout the morning into the evening. This is consistent with the overprediction of low ozone values evidenced in Fig. 3. At 1900 EDT, approximately an hour before sunset, the mean bias begins to increase again to the levels found in the early morning. The exception to this pattern is Beltsville (Fig. 4b) where the bias remains fairly consistent throughout the day.

The extent to which these biases are significant varies from site to site. The bias at Beltsville for both models is significant for 23 hours of the day, losing significance at 2100 EDT. Aldino (Fig. 4a) is the only site to display a significant negative bias for both models, occurring between 1800 EDT and 1900 EDT. The diurnal pattern at Fairhill and Padonia (Figs. 4e, f respectively) closely resembles that of Aldino, only positively shifted by approximately 7 ppbv and 10 ppbv, respectively, throughout the morning and afternoon. This yields significant positive differences between 0000 EDT 1100 EDT at both locations. Both Edgewood and Essex (Fig. 4c, d) exhibit a sharp decrease in bias at 1900 EDT followed by a sharp increase the following hour. The proximity of these two sites to the bay exposes the monitors to bay-breeze circulations which are known to cause a spike in ozone at approximately this time (Stauffer et al., *this issue*).

The RMSE exhibits few common features among the sites. The average RMSE tends to be greater in the afternoon (approximately 22 ppbv) compared to the morning (approximately 15 ppbv) with the exception of Beltsville, Padonia, and (to a lesser extent) Fairhill with fairly constant RMSE throughout the day. The variability in the RMSE for any given hour, evidenced by the CI, is small at Fairhill and Padonia relative to the other sites with CI of approximately 7–10 ppbv. In contrast, the greatest variability for any given hour is found at Edgewood and Essex with CI of approximately 10 - 20 ppbv. This hourly variability at Aldino is low throughout most of the day except in the afternoon (1400–2000 EDT) where the CI more than doubles from 7 ppbv to 15 ppbv. Similar spikes in CI occur in the NAQFC-*β* at Edgewood and Essex after 2100 EDT. These spikes seem to coincide with periods during or shortly after large changes in the bias, though this is not true for relatively smaller spikes at other sites. This is likely due to the models incorrectly forecasting these late-day transition periods.

**Fig. 5:**
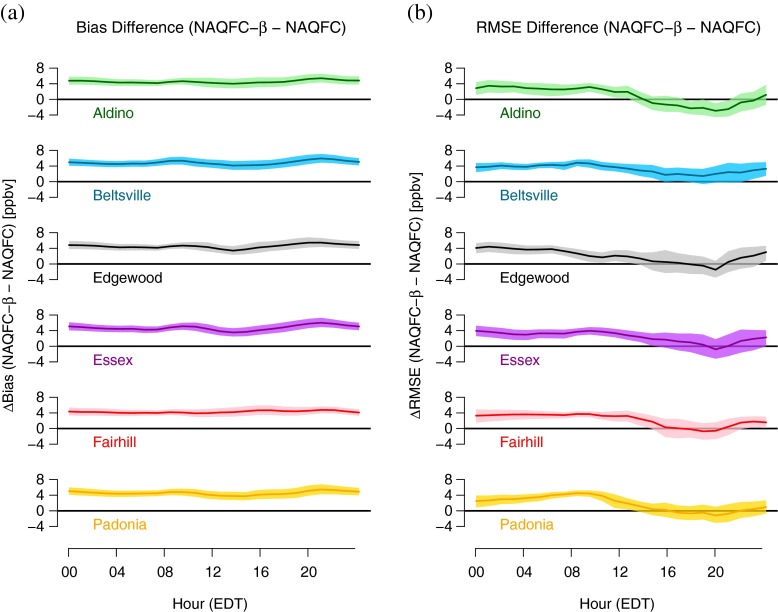
The difference in **a** the bias and **b** the RMSE between the NAQFC and NAQFC-*β* using the NAQFC as the reference value (NAQFC-*β* −NAQFC)

Figure 5a depicts the differences in the bias between the NAQFC and NAQFC-*β* as a function of hour of the day. The NAQFC-*β* consistently forecasts 1-h surface ozone 4 ppbv greater than the NAQFC throughout the day. There are slight inflections in the bias difference at all the sites at 0900 EDT, 1300 EDT, and 2100 EDT. These inflections are most pronounced at Beltsville, Edgewood, and Essex; however these inflections constitute changes in the bias on orders of less than 1 ppbv.

Figure 5b is the difference in RMSE between the two models at the six sites as a function of hour of the day. There is more diurnal variability in the RMSE differences when compared to the bias. Generally, the difference in RMSE between the two models decreases throughout the day, minimizing in the late afternoon, and rising back to pre-dawn levels after 2000 EDT. The exception is Padonia which starts the day low and peaks at 0900 EDT before becoming similar to the other sites. The CI generally increases into the afternoon hours at all sites except Aldino which stays fairly constant throughout the day.

### Value of information

**Fig. 6:**
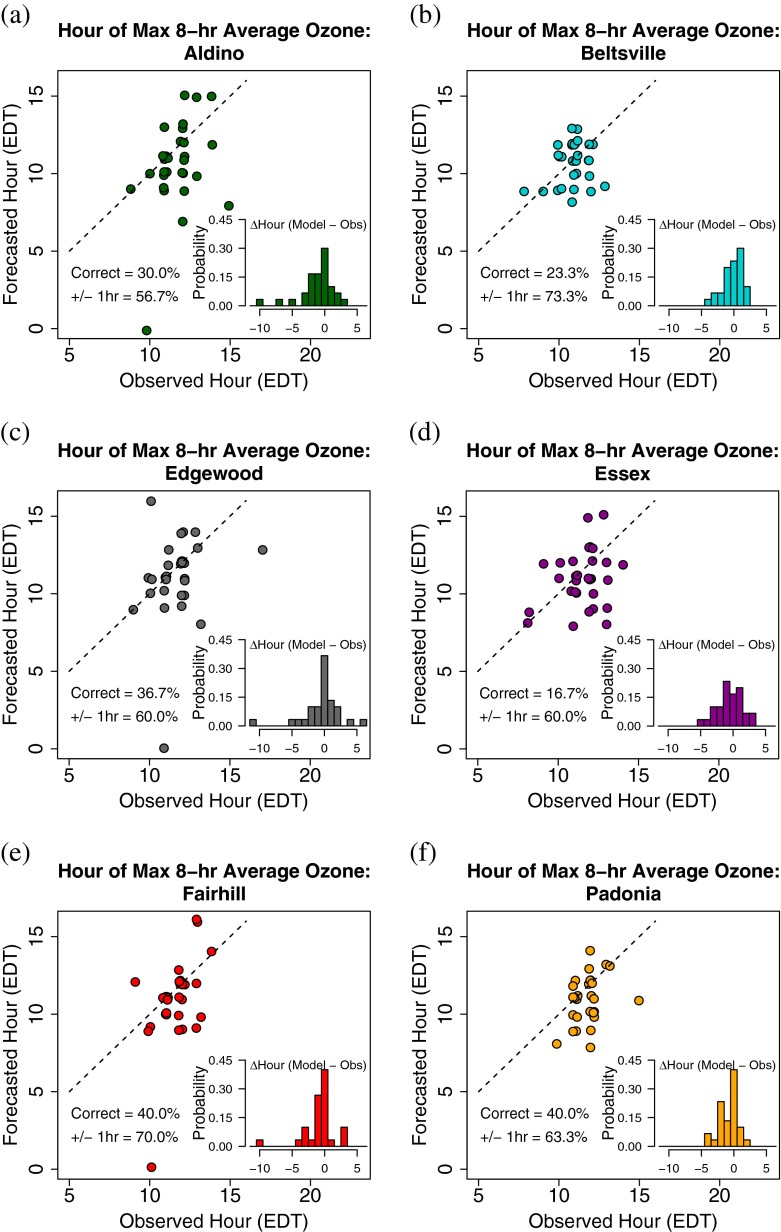
Scatterplot of the hour (EDT) at which the maximum 8-h average ozone was forecasted versus when it was observed at **a** Aldino, **b** Beltsville, **c** Edgewood, **d** Essex, **e** Fairhill, and **f** Padonia. The points are jittered slightly so that multiple points at the same coordinates are easily viewable. A dashed 1:1 line is provided for clarity. A histogram depicts the frequency of the difference between the forecasted and observed hour of maximum 8-h average ozone expressed as a probability

The maximum forecasted 8-h average ozone does not always coincide with when the maximum is actually observed. To analyze this, a scatterplot of the hours at which the maximum 8-h average ozone was forecasted and observed is shown in Fig. 6. Histograms of the difference between the forecasted and observed hour of maximum 8-h average ozone are provided in the lower-right of each plot.

Both models produced the same hours of maximum ozone for each day at all sites, so the forecasted hour in Fig. 6 refers to both the NAQFC and NAQFC-*β*. Generally, the models do well at forecasting the hour of maximum 8-h average ozone. The models forecast the hour correctly between 16.7 % (Essex) and 40.0 % (Fairhill and Padonia) of the time while forecasting within one hour of the maximum between 56.7 % (Aldino) and 73.3 % (Beltsville) of the time. The distribution of the difference in forecasted and observed hours of maximum 8-h average ozone is biased slightly low with means ranging from −0.17 (Beltsville) to −1.10 (Aldino) and medians of −0.5 (Aldino and Essex) or zero. This is to be expected because the 1-h forecasted ozone at all sites starts off with a high bias in the late morning that decreases into the afternoon (Fig. 4). When producing the running average, the hour at which the maximum occurs in the model will be skewed towards the time with the greater bias relative to the observations.

There is one outlier at Aldino, Edgewood, and Fairhill where the forecasted hour of maximum 8-h average ozone is at hour 0000 EDT. These points are all associated with 08 July. The model runs erroneously put a large cloud fraction over the northern half of the DISCOVER-AQ campaign area (not shown), keeping afternoon ozone production low and thus yielding a maximum 8-h average ozone that occurs at the start of the day. Scattered afternoon cloud cover allowed enough actinic flux to produce an ozone peak before becoming overcast and precipitating.

**Fig. 7:**
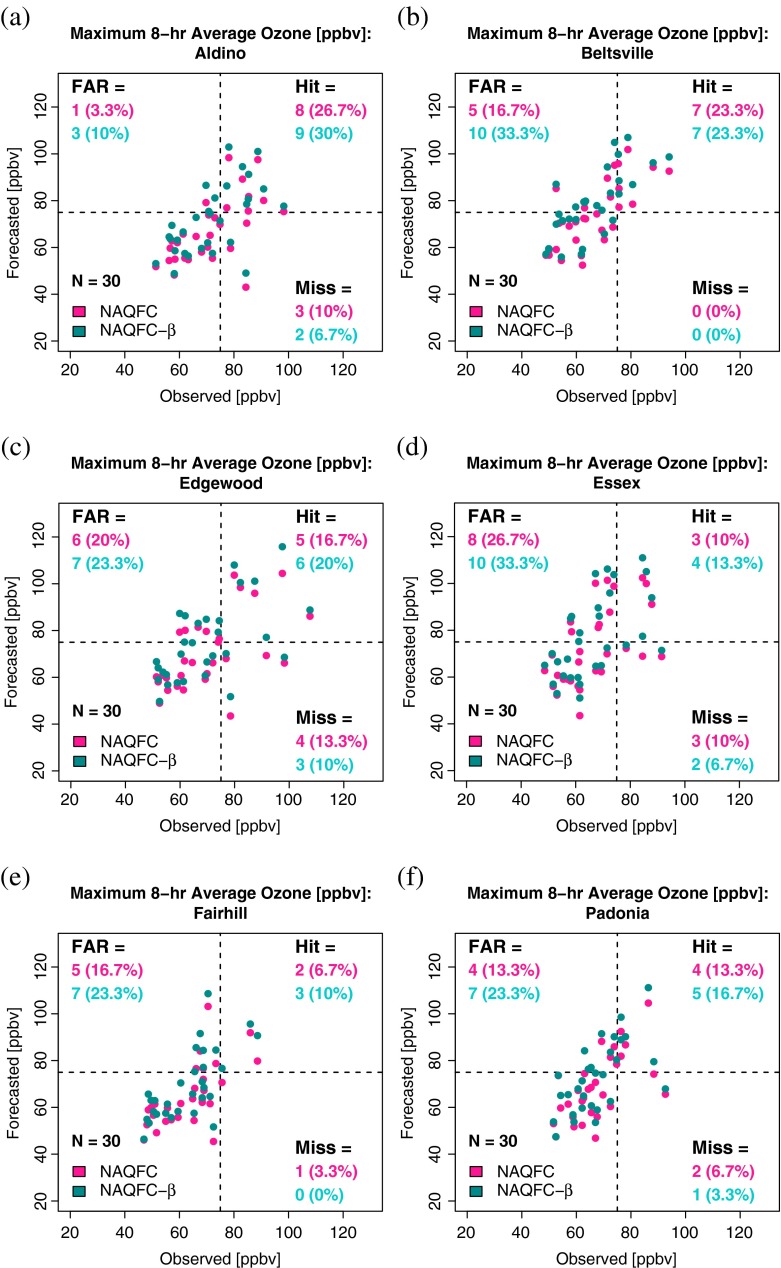
Summary of the general skill of the NAQFC (magenta) and the NAQFC-*β* (cyan) in forecasting maximum daily 8-h average surface ozone at **a** Aldino, **b** Beltsville, **c** Edgewood, **d** Essex, **e** Fairhill, and **f** Padonia. The dashed lines indicate the current NAAQS standard of 75 ppbv for an 8-h average ozone mixing ratio. The false alarm rate (FAR), hit rate (Hit), and miss rate (Miss) are provided as the number of observation-forecast pairs and corresponding percentage in parentheses

Scatterplots of the daily maximum 8-h average ozone are shown in Fig. 7.

The hit rates, miss rates, and false alarm rates are provided in their respective quadrants in each plot. The differences in these rates between the two models exhibit the behavior one would expect when analyzing two similar yet unequally biased models. The NAQFC-*β*, which was shown in Fig. 5a to have a significant positive difference in bias relative to the NAQFC, generally produces more hits and false alarms while reducing misses. In Beltsville, where the NAQFC fails to register a missed forecast, the higher biased NAQFC-*β* registers only an increase in false alarms.

**Fig. 8:**
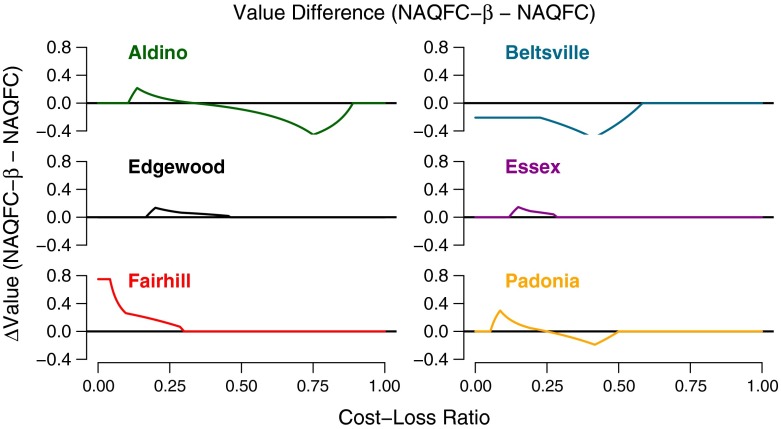
Relative difference in value between the NAQFC and the NAQFC-*β* as a function of the cost-loss ratio

The small differences in the hits, misses, and false alarms between the two models have large impacts on the value of information to the end user. Figure 8 is the difference in value of information between the NAQFC and NAQFC-*β* as a function of cost-loss ratio.

The higher biased NAQFC-*β*, relative to the NAQFC, tends to convert “missed” forecasts into “hit” forecasts. This effectively increases the maximum relative value of the NAQFC-*β* while simultaneously reducing the minimum cost-loss ratio at which the NAQFC-*β* produce value. Coincidently, the increased false alarm rate tends to reduce the maximum cost-loss ratio at which the NAQFC-*β* produces value (Wandishin and Brooks 2002).

The most dramatic differences are at Aldino, Beltsville, Fairhill, and Padonia. The differences in value at Aldino and Padonia switch from positive at low cost-loss ratios to negative at high cost-loss ratios. This indicates that both the NAQFC and the NAQFC-*β* must be used together at these two sites to optimize the value over a broad range of decision scenarios. The NAQFC provides greater forecast value at Beltsville through a cost-loss ratio of 0.61 at which point both models produce equal value. The NAQFC-*β* produces greater forecast value at Fairhill through a cost-loss ratio of 0.30 before the difference in value between the models becomes negligible. The NAQFC-*β* provides slightly greater forecast value than the NAQFC at the Edgewood and Essex sites, though this difference is small and over a short range of cost-loss ratios.

## Summary and Conclusions

Two numerical air quality models provided forecast support in flight decisions during the 2011 DISCOVER-AQ campaign. The updated chemical mechanism in the NAQFC-*β* tends to produce higher surface ozone mixing ratios than the mechanism used in the NAQFC (Saylor and Stein 2012). Statistical tests were performed to evaluate the skill of these two numerical models in predicting surface ozone, to determine the statistical significance of any differences in surface ozone predicted by the two models, and to assess the change in the value of information as a result of the updated mechanism.

A domain-wide analysis of the differences in 24-h forecasted surface ozone revealed significant differences between the two models. The MDI indicates that the NAQFC-*β* is at least 3.51 ppbv higher than the NAQFC. These background differences are fairly homogeneous throughout the domain during the early morning hours with a few maxima associated with the locations of known emissions sources standing out as slightly greater than the mean. Both the mean and the spread of these differences increase in the afternoon primarily in the southern portions of the model domain and over the Atlantic ocean. The regional nature of the elevated MDI fades into the evening hours revealing MDI maxima up to 6 ppbv located over emissions sources. These results confirm that the CB05 chemical mechanism in the NAQFC-*β* produces statistically significant differences in the predicted surface ozone compared to the NAQFC.

The skill of these two models were analyzed at the six surface sites of interest in the DISCOVER-AQ campaign. The standard descriptive statistics indicate that the NAQFC outperforms the NAQFC-*β*. The correlation between the NAQFC and surface observations were either identical or slightly better than the correlation between the NAQFC-*β* and surface observations. The NAQFC also provided predictions with significantly less bias and significantly less error than the NAQFC-*β* at all six sites. Both models typically overpredict surface ozone in low ozone regimes (0–30 ppbv) while tending to underpredict in high ozone regimes (75–90 ppbv).

The bias diurnal pattern for both models is consistent throughout the day among all of the sites. The bias is significantly high in the morning hours up until the hours of maximum ozone production at which time the biases typically drop to insignificant levels. The only exceptions to this are Beltsville, where the bias is significantly high throughout the day, and Aldino, which is the only site in which the bias becomes significantly negative in the afternoon. The CIs about the mean biases are fairly stable throughout the day, only slightly increasing in the afternoon hours at all the sites.

The RMSE diurnal patterns have few common features among all the sites. The RMSE typically decreases throughout the morning hours until approximately 1300 EDT when the RMSE begins to rise. Beltsville, like in the bias diurnal patterns, is fairly constant throughout the day. Padonia exhibits a fairly constant RMSE diurnal profile with the largest variability occuring in the morning according to the CI. The CI at the other sites tends to start off small and increase in the afternoon and evening hours. This increase in variability in RMSE can be sharp at sites including Aldino, Edgewood, and Essex.

The bias is significantly higher in the NAQFC-*β* than the NAQFC throughout the enitre day. Slight perturbations in the diurnal pattern occur at the ozone transition times, though these account for less than a 1 ppbv variation in the difference in the bias. The differences in the RMSE tend to start high in the morning hours and decrease to insignificant differences in the afternoon. The only exception is Beltsville where the NAQFC-*β* remains significantly high all day.

The descriptive statistics indicate that the NAQFC performs significantly better than the NAQFC-*β* in predicting surface ozone mixing ratios; however, the utility of the model is dependent on the needs of the end user. A static cost-loss ratio model was used to assess the relative difference in the value each of these models provide the user. The NAQFC-*β* produced greater value of information, typically at low cost-loss ratios, relative to the NAQFC. Beltsville was the only site in this analysis where the NAQFC provides more value than the NAQFC-*β*. Aldino and Padonia are the only sites where a combination of both models would yield the best overall value in decision-making. This is counter-intuitive, but the less skillful model produces greater value of information than the more skillful model for certain decisions. This is because standard evaluation metrics often mask the sensitivity of the end users’ needs to forecast error.
